# Mucosal boosting enhances vaccine protection against SARS-CoV-2 in macaques

**DOI:** 10.1038/s41586-023-06951-3

**Published:** 2023-12-14

**Authors:** Katherine McMahan, Frank Wegmann, Malika Aid, Michaela Sciacca, Jinyan Liu, Nicole P. Hachmann, Jessica Miller, Catherine Jacob-Dolan, Olivia Powers, David Hope, Cindy Wu, Juliana Pereira, Tetyana Murdza, Camille R. Mazurek, Amelia Hoyt, Adrianus C. M. Boon, Meredith Davis-Gardner, Mehul S. Suthar, Amanda J. Martinot, Mona Boursiquot, Anthony Cook, Laurent Pessaint, Mark G. Lewis, Hanne Andersen, Jeroen Tolboom, Jan Serroyen, Laura Solforosi, Lea M. M. Costes, Roland C. Zahn, Dan H. Barouch

**Affiliations:** 1 Center for Virology and Vaccine Research, Beth Israel Deaconess Medical Center, Boston, MA USA; 2 Janssen Vaccines and Prevention, Leiden, Netherlands; 3 Ragon Institute of MGH, MIT and Harvard, Cambridge, MA USA; 4 Washington University School of Medicine, St Louis, MO USA; 5 Emory School of Medicine, Atlanta, GA USA; 6 Tufts University Cummings School of Veterinary Medicine, Grafton, MA USA; 7 Bioqual, Rockville, MD USA

**Keywords:** Vaccines, Mucosal immunology

## Abstract

A limitation of current SARS-CoV-2 vaccines is that they provide minimal protection against infection with current Omicron subvariants^1,2^, although they still provide protection against severe disease. Enhanced mucosal immunity may be required to block infection and onward transmission. Intranasal administration of current vaccines has proven inconsistent^3–7^, suggesting that alternative immunization strategies may be required. Here we show that intratracheal boosting with a bivalent Ad26-based SARS-CoV-2 vaccine results in substantial induction of mucosal humoral and cellular immunity and near-complete protection against SARS-CoV-2 BQ.1.1 challenge. A total of 40 previously immunized rhesus macaques were boosted with a bivalent Ad26 vaccine by the intramuscular, intranasal and intratracheal routes, or with a bivalent mRNA vaccine by the intranasal route. Ad26 boosting by the intratracheal route led to a substantial expansion of mucosal neutralizing antibodies, IgG and IgA binding antibodies, and CD8^+^ and CD4^+^ T cell responses, which exceeded those induced by Ad26 boosting by the intramuscular and intranasal routes. Intratracheal Ad26 boosting also led to robust upregulation of cytokine, natural killer, and T and B cell pathways in the lungs. After challenge with a high dose of SARS-CoV-2 BQ.1.1, intratracheal Ad26 boosting provided near-complete protection, whereas the other boosting strategies proved less effective. Protective efficacy correlated best with mucosal humoral and cellular immune responses. These data demonstrate that these immunization strategies induce robust mucosal immunity, suggesting the feasibility of developing vaccines that block respiratory viral infections.

## Abstract (web summary)

Intratracheal boosting with a bivalent Ad26-based SARS-CoV-2 vaccine results in substantial induction of mucosal humoral and cellular immunity and near-complete protection against SARS-CoV-2 BQ.1.1 in rhesus macaques.

## Main

Current SARS-CoV-2 vaccines provide moderate efficacy against severe disease^8,9^ but minimal to no efficacy against the acquisition of infection with Omicron subvariants^1,2^. A key problem is that intramuscular immunization with mRNA and adenovirus-vector-based SARS-CoV-2 vaccines does not typically induce robust mucosal immunity^10,11^. The coronavirus vaccine roadmap^12^ and Project NextGen^13^ emphasize the need to develop next-generation SARS-CoV-2 vaccines and vaccination strategies that induce improved mucosal immunity that will block infection and onward transmission. However, the development of immunization strategies that induce consistent and robust mucosal immune responses has proven difficult and may require innovative approaches that are beyond intranasal administration of current vaccines^3–5^. Here we compare multiple strategies to induce mucosal humoral and cellular immune responses in macaques.

## Study design

**Fig. 1:**
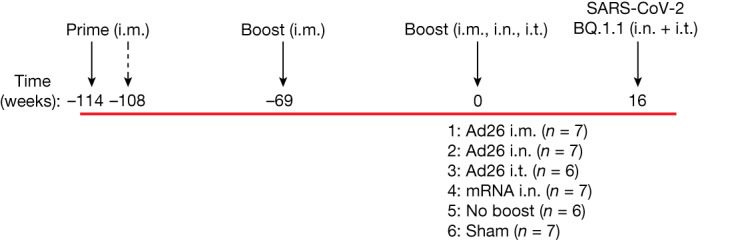
Study outline. A total of 40 rhesus macaques previously received one or two i.m. primes with Ad26.COV2.S at weeks −114 and −108 and one boost i.m. with either Ad26.COV2.S or Ad26.COV2.S.351 (Beta) at week −69. At week 0, macaques were boosted with the bivalent Ad26.COV2.S + Ad26.COV2.S.529 (Omicron BA1) vaccine by the i.m., i.n. or i.t. routes or the bivalent mRNA vaccine (Pfizer-BioNTech) by the i.n. route. Another group received no boost at week 0, and a naive sham control group was included. At study week 16, all of the macaques were challenged with SARS-CoV-2 BQ.1.1 through the i.n. and i.t. routes.

Forty adult rhesus macaques were primed with one or two doses of the Ad26.COV2.S vaccine (Janssen/Johnson & Johnson)^14–17^ by the intramuscular (i.m.) route at week −114 and week −106 and were boosted with either the Ad26.COV2.S or Ad26.COV2.S.351 vaccine^18^ by the i.m. route at week −69 (Fig. 1). Humoral and cellular immune responses in these animals after initial priming and boosting have been described previously^18^. Macaques that received two or three immunizations were equally divided into subsequent boosting groups for the present study. At week 0, reflecting approximately annual boosting, macaques were boosted with 5 × 10^10^ viral particles of the bivalent Ad26.COV2.S + Ad26.COV2.S.529 vaccine^19^ by the i.m., intranasal (i.n.) or intratracheal (i.t.) routes or 30 μg of the lipid nanoparticle formulated bivalent mRNA vaccine (Pfizer-BioNTech)^20–22^ by the i.n. route (*n* = 6–7 macaques per group) (Fig. 1). Another group received no boost at week 0, and a sham control group was included for the challenge phase of the study. At study week 16, all of the macaques were challenged with a high dose of 2 × 10^6^ plaque-forming units (PFU) of SARS-CoV-2 BQ.1.1 by the i.n. and i.t. routes.

## Mucosal and peripheral humoral responses

**Fig. 2:**
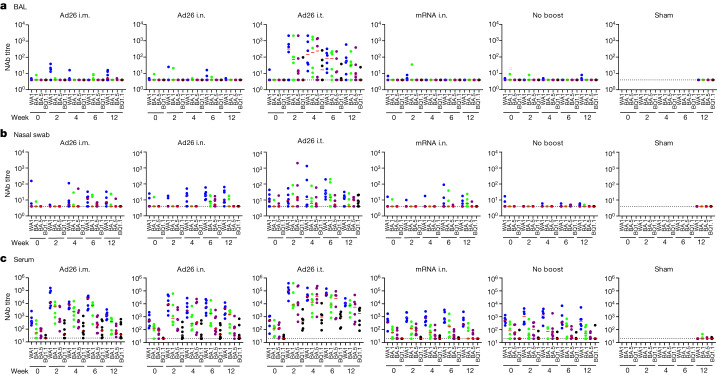
Mucosal and peripheral SARS-CoV-2 NAb responses. **a**–**c**, NAb titres were assessed before and after boosting using a luciferase-based pseudovirus neutralization assay in BAL (**a**), nasal swabs (**b**) and serum (**c**). Responses were measured against SARS-CoV-2 WA1/2020 (blue), BA.1 (green), BA.5 (purple) and BQ.1.1 (black). The missing symbols indicate the absence of data. The dotted lines represent the limits of quantification. The median (red bars) values are shown. *n* = 40 biologically independent macaques. Wk, week. Source Data

**Extended Data Fig. 1:**
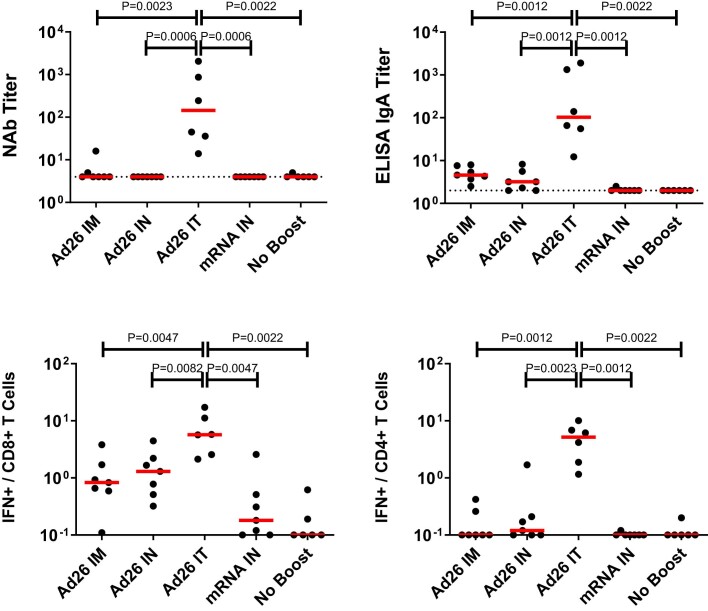
Comparison of week 4 immune responses in BAL. Comparison of NAb, IgA, CD8^+^ T cell responses, and CD4^+^ T cell responses in BAL across groups at week 4 (Figs. 2–4). NAb and IgA responses to WA1/2020 and CD8^+^ and CD4^+^ T cell responses to BA.5 are shown. These immunogenicity comparisons were secondary objectives of the study; groups were compared by two-sided Mann-Whitney tests and unadjusted P values are shown. n = 40 biologically independent animals.

Mucosal and peripheral antibody responses were assessed in bronchoalveolar lavage (BAL) fluid, nasal swab eluate and serum, both before and after boosting. Neutralizing antibody (NAb) titres were assessed using luciferase-based pseudovirus-neutralization assays in each anatomical compartment^23^. In BAL, low and sporadic NAb responses were observed at week 0 before boosting and at weeks 2, 4, 6 and 12 after boosting in the Ad26 i.m., Ad26 i.n., mRNA i.n. and no-boost groups (Fig. 2a). By contrast, robust NAb responses were observed in BAL after boosting in the Ad26 i.t. group. Median NAb titres in the BAL in the Ad26 i.t. group were 145, 193, 236 and 17 at week 4, and 53, 32, 8 and 4 at week 12 to WA1/2020, BA.1, BA.5 and BQ.1.1, respectively (Fig. 2a). WA1/2020 NAb titres in the BAL were higher in the Ad26 i.t. group compared with in the Ad26 i.m., Ad26 i.n. and mRNA i.n. groups at week 4 (*P* = 0.0006 to *P* = 0.0023, two-sided Mann–Whitney *U*-tests; Extended Data Fig. 1). NAb responses in nasal swabs were generally low, except in the Ad26 i.t. group. Median NAb titres in nasal swabs to WA1/2020 were 12, 19 and 26 at week 6 in the Ad26 i.m., i.n. and i.t. groups, respectively (Fig. 2b). NAb responses in serum were observed in all of the immunized groups at week 0 before boosting, consistent with the reported long-term memory of serum NAbs after Ad26 i.m. immunization^18,24^ (Fig. 2c). Median NAb titres in the serum at week 12 were 1,241, 388, 53 and 39 in the Ad26 i.m. group; 4,159, 1,530, 133 and 28 in the Ad26 i.n. group; and 11,163, 7,683, 5,506 and 1,738 in the Ad26 i.t. group to WA1/2020, BA.1, BA.5 and BQ.1.1, respectively (Fig. 2c).

**Fig. 3:**
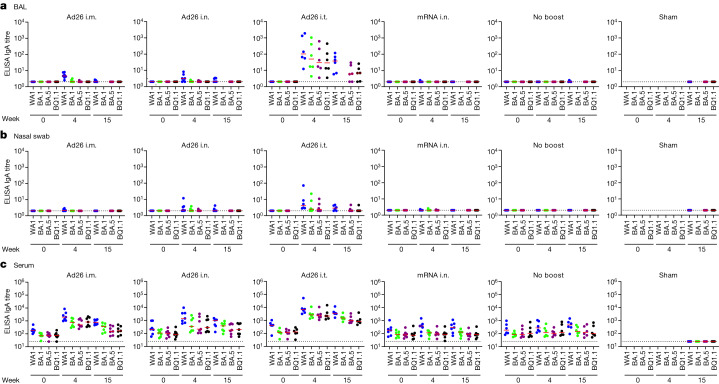
Mucosal and peripheral IgA spike-specific binding antibody responses. **a**–**c**, IgA spike-specific binding antibody responses were assessed before and after boosting using ELISA in BAL (**a**), nasal swabs (**b**) and serum (**c**). Responses were measured against SARS-CoV-2 WA1/2020 (blue), BA.1 (green), BA.5 (purple) and BQ.1.1 (black) spike proteins. The missing symbols indicate the absence of data. The dotted lines represent the limits of quantification. Median (red bars) values are shown. *n* = 40 biologically independent macaques. Source Data

**Extended Data Fig. 2:**
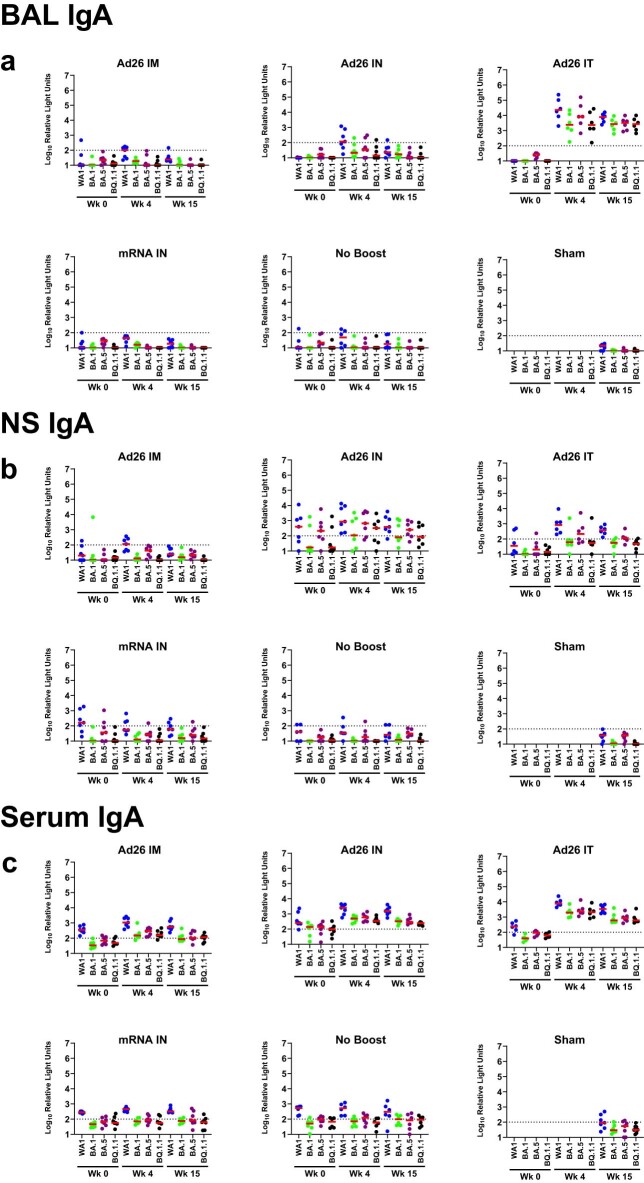
Mucosal and peripheral IgA spike-specific binding antibody responses by ECLA. IgA spike-specific binding antibody responses were assessed before and after boosting by meso-scale discovery (MSD) electrochemoluminscent assay in (**a**) bronchoalveolar lavage (BAL), (**b**) nasal swabs (NS), and (**c**) serum. Responses were measured against SARS-CoV-2 WA1/2020 (blue), BA.1 (green), BA.5 (purple), and BQ.1.1 (black) spike proteins. Missing symbols indicate absence of data. Dotted lines represent limits of quantitation. Medians (red bars) are shown. Relative light units are shown. n = 40 biologically independent animals.

**Extended Data Fig. 3:**
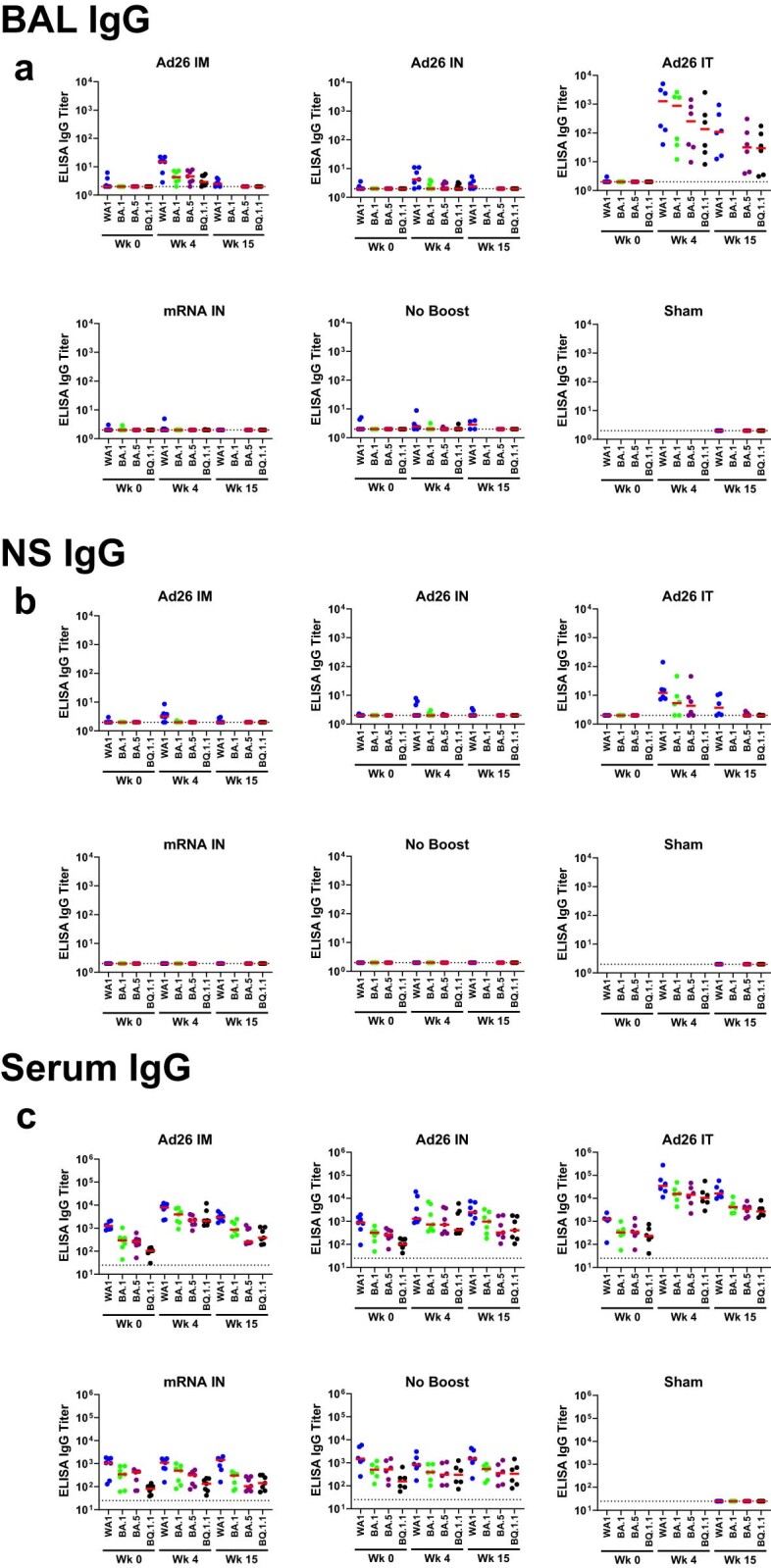
Mucosal and peripheral IgG spike-specific binding antibody responses by ELISA. IgG spike-specific binding antibody responses were assessed before and after boosting by ELISA in (**a**) bronchoalveolar lavage (BAL), (**b**) nasal swabs (NS), and (**c**) serum. Responses were measured against SARS-CoV-2 WA1/2020 (blue), BA.1 (green), BA.5 (purple), and BQ.1.1 (black) spike proteins. Missing symbols indicate absence of data. Dotted lines represent limits of quantitation. Medians (red bars) are shown. n = 40 biologically independent animals.

**Extended Data Fig. 4:**
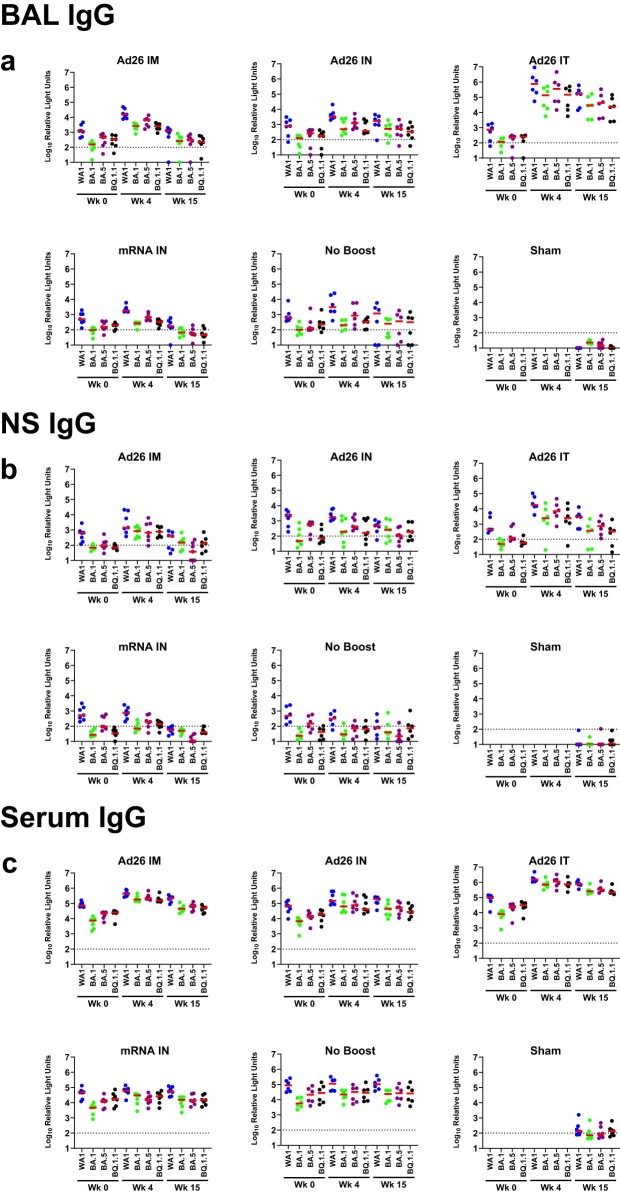
Mucosal and peripheral IgG spike-specific binding antibody responses by ECLA. IgG spike-specific binding antibody responses were assessed before and after boosting by meso-scale discovery (MSD) electrochemoluminscent assay in (**a**) bronchoalveolar lavage (BAL), (**b**) nasal swabs (NS), and (**c**) serum. Responses were measured against SARS-CoV-2 WA1/2020 (blue), BA.1 (green), BA.5 (purple), and BQ.1.1 (black) spike proteins. Missing symbols indicate absence of data. Dotted lines represent limits of quantitation. Medians (red bars) are shown. Relative light units are shown. n = 40 biologically independent animals.

SARS-CoV-2 spike-specific binding antibody responses were assessed using enzyme-linked immunosorbent assays (ELISAs) and electrochemiluminescence assays (ECLAs)^25^. In BAL, no IgA responses as determined by ELISA were observed at week 0 before boosting and minimal IgA responses were observed at weeks 4 and 15 after boosting in the Ad26 i.m., Ad26 i.n., mRNA i.n. and no-boost groups, but median IgA titres in BAL in the Ad26 i.t. group were 103, 50, 30 and 29 at week 4 to WA1/2020, BA.1, BA.5 and BQ.1.1, respectively (Fig. 3a and Extended Data Fig. 1). IgA titres as determined by ELISA in nasal swabs and serum were also detected most prominently in the Ad26 i.t. group (Fig. 3b,c). IgA responses as determined by ECLA (Extended Data Fig. 2) and IgG responses as determined by ELISA and ECLA showed similar trends (Extended Data Figs. 3 and 4), with the most robust responses in the Ad26 i.t. group. These data demonstrate that Ad26 boosting by the i.t. route led to substantial increases in both mucosal and peripheral NAbs as well as IgA and IgG binding antibodies.

## Mucosal and peripheral T cell responses

**Fig. 4:**
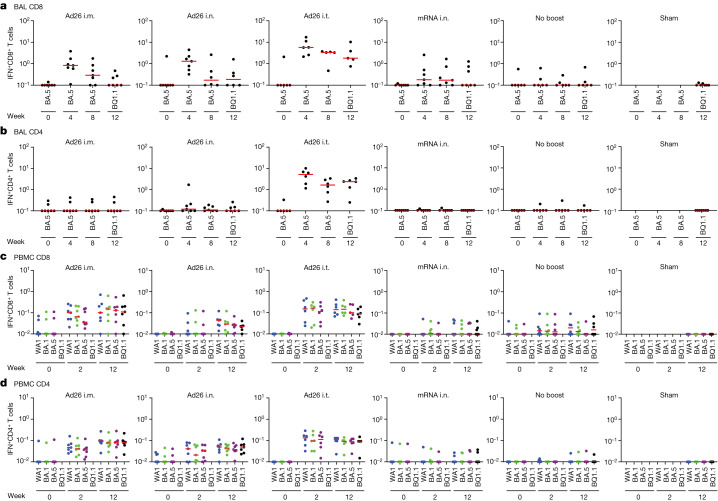
Mucosal and peripheral T cell responses. **a**–**d**, Pooled peptide spike-specific IFNγ CD8^+^ (**a**,**c**) and CD4^+^ (**b**,**d**) T cell responses as the percentage of total CD8^+^ and CD4^+^ T cells, respectively, were assessed before and after boosting by intracellular cytokine staining assays in BAL cells (**a** and **b**) and PBMCs (**c** and **d**). Responses were measured against SARS-CoV-2 BA.5 or BQ.1.1 spike peptides due to limited numbers of BAL cells (**a**,**b**) or WA1/2020 (blue), BA.1 (green), BA.5 (purple) and BQ.1.1 (black) spike peptides (**c**,**d**). The missing symbols indicate the absence of data. Median (red bars) values are shown. *n* = 40 biologically independent macaques. Source Data

**Extended Data Fig. 5:**
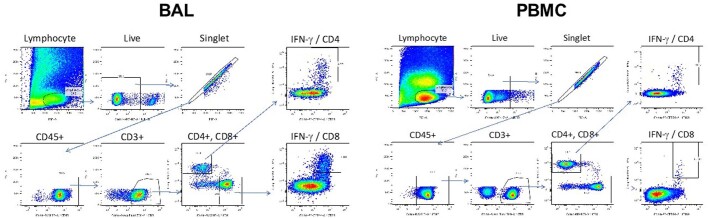
Sample flow cytometry gating. Intracellular cytokine staining gating analysis for CD4^+^ and CD8^+^ T cells in BAL cells (left) and PBMCs (right).

Mucosal and peripheral spike-specific IFNγ^+^CD8^+^ and CD4^+^ T cell responses were assessed by intracellular cytokine staining^26^ in BAL cells and peripheral blood mononuclear cells (PBMCs) (Extended Data Fig. 5). Median mucosal CD8^+^ T cell responses in BAL were largely negative at week 0 before boosting but increased to 0.83%, 1.30% and 5.72% against BA.5 at week 4 and to <0.10%, 0.19% and 1.80% against BQ.1.1 at week 12 in the Ad26 i.m., i.n. and i.t. groups, respectively (Fig. 4a). Mucosal CD8^+^ T cell responses in BAL increased marginally in the mRNA i.n. group and not at all in the no-boost group (Fig. 4a). Median mucosal CD4^+^ T cell responses in BAL increased markedly to 5.17% against BA.5 at week 4 and to 2.38% against BQ.1.1 at week 12 in the Ad26 i.t. group, but were not enhanced in the other groups (Fig. 4b). Mucosal CD8^+^ and CD4^+^ T cell responses in BAL were higher in the Ad26 i.t. group compared with in the Ad26 i.m., Ad26 i.n. and mRNA i.n. groups (*P* = 0.0012 to *P* = 0.0082; Extended Data Fig. 1). Peripheral CD8^+^ and CD4^+^ T cell responses in PBMCs increased after boosting in the Ad26 i.m., i.n. and i.t. groups but were not increased in the mRNA i.n. or no-boost groups (Fig. 4c,d). These data demonstrate that Ad26 boosting by the i.t. route led to substantial increases in mucosal and peripheral CD8^+^ and CD4^+^ T cell responses.

## Protective efficacy

**Fig. 5:**
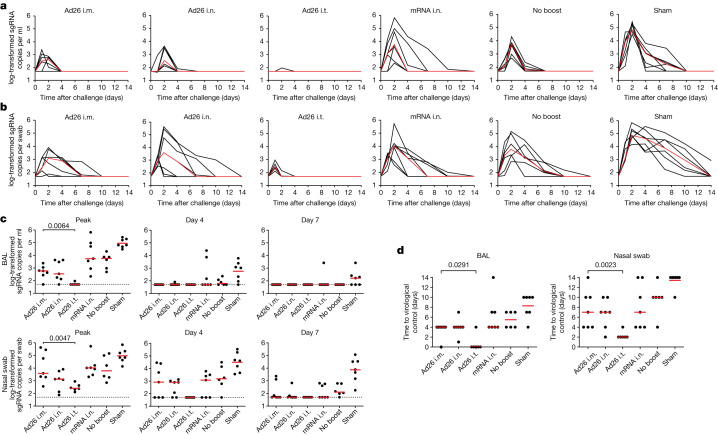
Viral loads after SARS-CoV-2 BQ.1.1 challenge. **a**,**b**, log-transformed sgRNA copies per ml in BAL (**a**) and log-transformed sgRNA copies per swab in nasal swabs (**b**) after SARS-CoV-2 BQ.1.1 challenge. Median (red lines) values are shown. **c**, log-transformed sgRNA copies per ml in BAL and nasal swabs at peak, day 4 and day 7 after SARS-CoV-2 BQ.1.1 challenge. **d**, The number of days to undetectable viral loads in the BAL and nasal swabs. The dotted lines represent the limits of quantification. Median (red bars) values are shown. The primary objective of the study was to compare the protective efficacy of Ad26 i.t. versus Ad26 i.m. boosting against SARS-CoV-2 challenge; these groups were therefore compared using two-sided Mann–Whitney *U*-tests and *P* values are shown. *n* = 40 biologically independent macaques. Source Data

At week 16, all of the macaques were challenged by the i.n. + i.t. routes with a with a high dose of 2 × 10^6^ PFU SARS-CoV-2 BQ.1.1 (hCoV-19/USA/CA-Stanford-106_S04/2022, EPI_ISL_15196219). To assess the protective efficacy, *E* subgenomic RNA (sgRNA) was quantified using quantitative PCR with reverse transcription (RT–qPCR) in BAL and nasal swabs after challenge^27^. The Ad26 i.t. group demonstrated near-complete protection, whereas the other vaccinated groups showed partial protection (Fig. 5). The median log-transformed sgRNA copies per ml in the BAL on day 2 was 2.63 (range, <1.70–2.87), 2.54 (<1.70–3.63), <1.70 (<1.70–1.97), 3.74 (2.34–5.82), 3.76 (2.69–4.31) and 4.81 (4.48–5.43) in the Ad26 i.m., Ad26 i.n., Ad26 i.t., mRNA i.n., no-boost and sham groups, respectively (Fig. 5a). The median log-transformed sgRNA copies per swab in nasal swabs on day 2 was 3.59 (range, <1.70–5.45), 3.09 (<1.70–3.91), 1.71 (<1.70–2.16), 4.02 (2.58–5.73), 3.79 (2.85–5.16) and 4.84 (4.14–5.85) in the Ad26 i.m., Ad26 i.n., Ad26 i.t., mRNA i.n., no-boost and sham groups, respectively (Fig. 5b).

Median peak sgRNA levels in BAL in the Ad26 i.t. group were >1.07 log lower than in the Ad26 i.m. group (*P* = 0.0064, two-sided Mann–Whitney *U*-test), >2.06 log lower than in the no boost group and >3.26 log lower than in the sham group (Fig. 5c). Median peak sgRNA levels in nasal swabs in the Ad26 i.t. group were 1.18 log lower than in the Ad26 i.m. group (*P* = 0.0047), 1.38 log lower than in the no-boost group and 2.57 log lower than in the sham group (Fig. 5c). Macaques in the Ad26 i.t. group also demonstrated more rapid resolution of viral replication in the BAL (*P* = 0.0291) and nasal swabs (*P* = 0.0023) compared with macaques in the Ad26 i.m. group (Fig. 5d).

**Extended Data Fig. 6:**
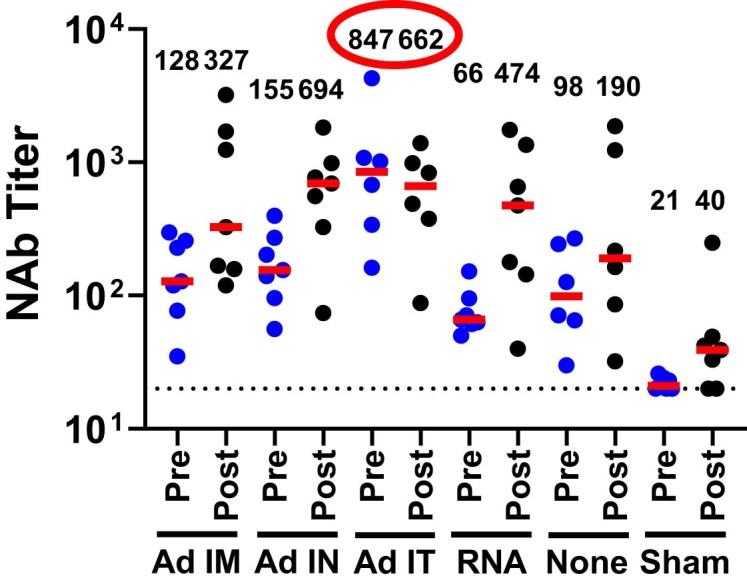
Anamnestic SARS-CoV-2 neutralizing antibody responses following SARS-CoV-2 BQ.1.1 challenge. Neutralizing antibody (NAb) titres to SARS-CoV-2 BQ.1.1 were assessed immediately before (Pre) and 2 weeks after (Post) challenge by a luciferase-based pseudovirus neutralization assay in serum. Dotted lines represent limits of quantitation. Medians (red bars) are shown with values numerically depicted. Note that all groups except the Ad26 IT group show increased BQ.1.1 NAb titres following challenge. n = 40 biologically independent animals.

In the Ad26 i.t. group, 5 out of 6 macaques showed no detectable virus in the BAL and rapid resolution of virus in nasal swabs by day 2 after challenge. Whether the low transient viral loads observed on day 1 in nasal swabs in the Ad26 i.t. group represented input challenge virus or limited viral replication is unclear^27^. Macaques in the Ad26 i.t. group also showed no anamnestic BQ.1.1 NAb responses after challenge, whereas all of the other groups showed a 2–7-fold increase in median BQ.1.1 NAb titres after challenge (Extended Data Fig. 6), suggesting exquisite virological control and near-complete or possibly complete protection in macaques in the Ad26 i.t. group.

**Extended Data Fig. 7:**
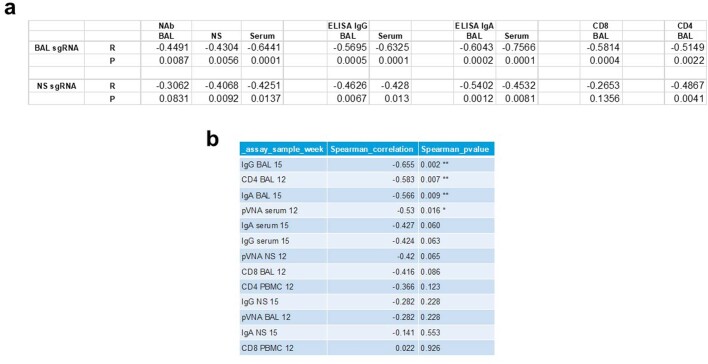
Immune correlates of protection. **a**, Correlation of pre-challenge, BQ.1.1-specific mucosal and peripheral NAb, ELISA IgG, ELISA IgA, and CD8^+^ and CD4^+^ T cell responses with peak log sgRNA levels in BAL and NS following SARS-CoV-2 BQ.1.1 challenge. Two-sided Spearman rank-correlation tests were performed, and R and P values are shown. **b**, Multivariable analysis of stepwise regression of peak sgRNA with immunologic parameters with multiple comparison adjustments.

We next assessed mucosal and peripheral immune correlates of protection after challenge. In a univariate analysis, BQ.1.1-specific NAb titres in the BAL, nasal swabs and serum, IgG titres in the BAL and serum, IgA titres in the BAL and serum, and CD8^+^ and CD4^+^ T cell responses in the BAL were inversely correlated with sgRNA levels in the BAL, and a subset of these parameters was also inversely correlated with sgRNA levels in nasal swabs (*P* = 0.0001 to *P* = 0.013, two-sided Spearman correlation tests) (Extended Data Fig. 7a). In a multivariate analysis involving only the Ad26-boosted groups, a stepwise regression of BQ.1.1-specific immune responses and peak sgRNA levels in the BAL showed that the strongest correlates of protection were the mucosal IgG, CD4^+^ T cell responses and IgA responses in the BAL (*P* = 0.002, *P* = 0.007 and *P* = 0.009, respectively, two-sided Spearman correlation tests) (Extended Data Fig. 7b). Peak sgRNA levels in the BAL also correlated with peak sgRNA levels in nasal swabs (*R* = 0.6912, *P* < 0.0001). These data suggest that the robust mucosal humoral and cellular immune responses after i.t. immunization contributed substantially to the protective efficacy observed in that group.

## Histopathology

**Extended Data Fig. 8:**
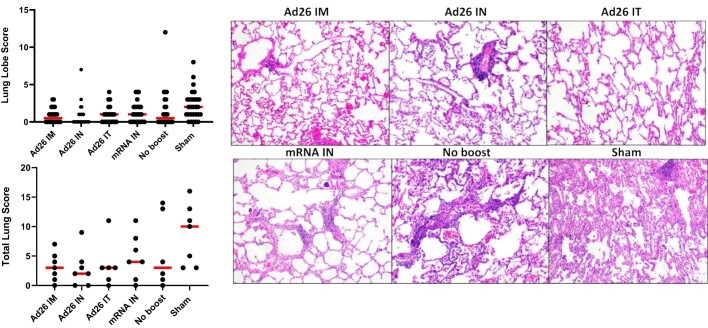
Histopathology. Individual lung lobe pathology scores (upper graph) and cumulative lung pathology scores (lower graph) for each macaque. Medians (red bars) are shown. Representative images are shown from lungs from each group 14 days following challenge. Sham vaccinated animals showed type II pneumocyte hyperplasia and expansion of the interstitium with inflammatory cells. Images are 10X magnification. n = 40 biologically independent animals.

**Extended Data Fig. 9:**
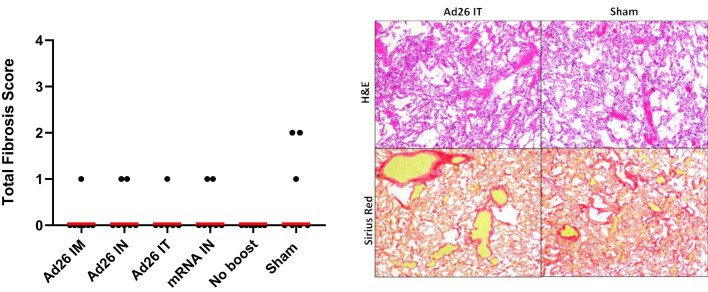
Fibrosis scores. Fibrosis sub-score from total lung score for individual macaques. Medians (red bars) are shown. Representative images are shown from lungs from an Ad26 IT and sham animal are shown with H&E and Sirius Red staining. Fibrosis was rare in all groups but slightly more evident in certain sham animals than vaccinated animals. Images are 10X magnification. n = 40 biologically independent animals.

Lungs were assessed for pathology at necropsy 14 days after challenge and were scored for residual interstitial inflammation, alveolar epithelial repair (type II pneumocyte hyperplasia) and fibrosis as previously described^28^. Minimal pathology was seen in all of the vaccinated groups at necropsy, although the sham controls showed more inflammation and higher pathology scores compared with the vaccinated groups (Extended Data Fig. 8). There was no histopathological evidence of pulmonary fibrosis (Extended Data Fig. 9) or other consistent histopathological findings in any of the vaccinated groups.

## Lung transcriptomics and cytokines

**Fig. 6:**
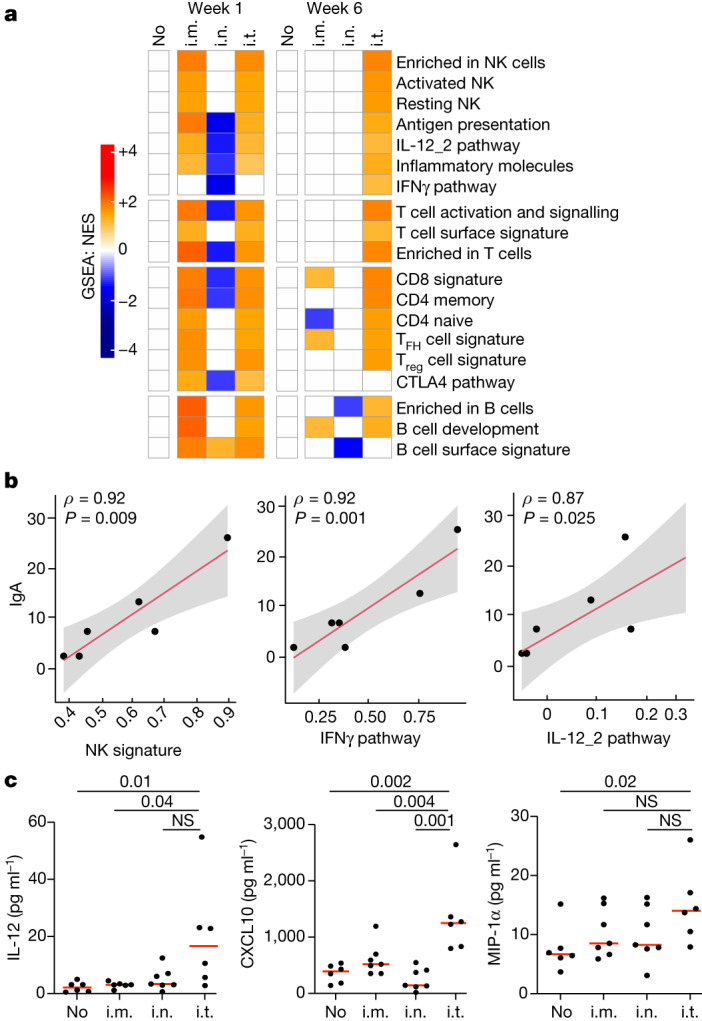
Transcriptomics and cytokine analyses in the BAL. **a**, Gene set enrichment analysis (GSEA) normalized enrichment scores (NES) of pathways for different groups at week 1 and week 6 after immunization relative to the no-boost (no) group. The colour scale represents the NES, ranging from downregulated pathways (blue) to no change (white) to upregulated pathways (orange). The colour intensity reflects the strength of the enrichment score, with darker colours indicating stronger upregulation (red) or downregulation (blue). Pathways were selected using a GSEA nominal *P* value of 0.05. *P* values were corrected for multiple testing using a false-discovery rate cut-off of 0.05. *n* = 40 biologically independent macaques. T_FH_, T follicular helper cells; T_reg_, regulatory T cells. **b**, Sample-level scores (sample-level enrichment analysis (SLEA)) for NK, IFNγ and IL-12_2 pathways at week 6 after i.t. boosting correlated with the IgA titres at week 12. Correlation analyses were performed using two-tailed Spearman correlation tests and simple linear regression. Lines of best fit (red line) and 95% confidence intervals (grey shading) are shown. *n* = 6 biologically independent macaques. **c**, IL-12, M1P-1a and CXCL10 levels in the BAL at week 6 were compared between groups using two-sided Mann–Whitney *U*-tests; *P* values are shown. *n* = 28 biologically independent macaques. NS, not significant. Source Data

**Extended Data Fig. 10:**
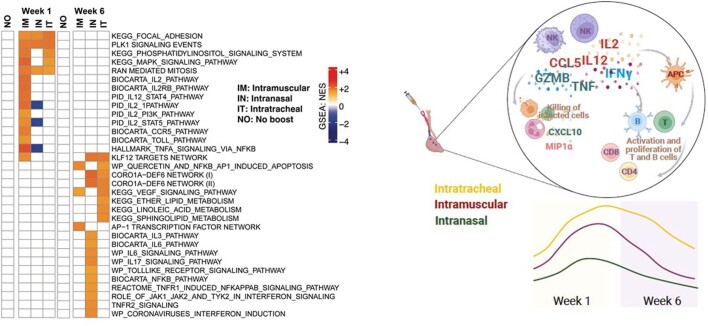
Additional transcriptomics analyses in the BAL. The heatmap (left) illustrates the normalized enrichment scores (NES) of pathways for different groups at weeks 1 and 6 following immunization relative to the no boost group. The colour scale represents the NES, ranging from downregulated pathways (blue) to no change (white) to upregulated pathways (orange). The colour intensity reflects the strength of the enrichment score, with darker colours indicating stronger upregulation or downregulation. Pathways were selected using a GSEA nominal P-value of 0.05. P-values were corrected for multiple testing using the false discovery rate FDR cutoff of 0.05. n = 40 biologically independent animals were included in this analysis. The summary cartoon (right) shows that IT boosting led to robust and sustained activation of cytokine, NK, T and B cell pathways in the lung for at least 6 weeks. The diagram was created using BioRender.

To investigate the mechanism of the potency of Ad26 i.t. boosting, we performed bulk RNA-sequencing analysis of BAL cells and cytokine analysis of BAL fluid at week 1 and week 6 after Ad26 i.m., i.n. and i.t. boosting and after no boost. Pathway enrichment analysis demonstrated upregulation of pathways associated with natural killer (NK) cell activation, antigen-presenting cell (APC) function, IFNγ and IL-12 signalling, and T and B cell activation at both week 1 and week 6 after i.t. boosting compared with no boost (Fig. 6a). By contrast, after i.m. boosting, these pathways were only transiently increased at week 1 but not at week 6 and, after i.n. boosting, these pathways were not induced at all (Fig. 6a). Moreover, NK, IFNγ and IL-12 signalling pathways in the BAL correlated with mucosal IgA responses after i.t. boosting (Fig. 6b), and proteomic cytokine analysis of the BAL fluid confirmed higher IL-12, MIP-1α and CXCL10 levels after i.t. boosting (Fig. 6c). i.t. boosting also led to increased pro-inflammatory and metabolic pathways in the lungs (Extended Data Fig. 10). Taken together, these data demonstrate that i.t. boosting led to robust and sustained activation of cytokine, NK, T and B cell pathways in the lungs for at least 6 weeks, which probably contributed to the mechanism through which i.t. boosting resulted in a robust and sustained enhancement of mucosal immunity and protective efficacy (Extended Data Fig. 10).

## Discussion

Our data demonstrate that Ad26 i.t. boosting robustly augmented mucosal humoral and cellular immune responses and provided near-complete protection against high-dose mucosal SARS-CoV-2 BQ.1.1 challenge in rhesus macaques. Ad26 i.t. boosting induced greater mucosal immunity compared with Ad26 i.n. and i.m. boosting for all of the immunological parameters evaluated. By contrast, mRNA i.n. boosting proved to be ineffective, suggesting that improved formulations will probably be required for effective mucosal delivery of mRNA vaccines. Notably, this mRNA vaccine was highly immunogenic in macaques when delivered through the i.m. route^29^. Mucosal humoral and cellular immune responses in BAL were the strongest immunological correlates of protection against mucosal SARS-CoV-2 challenge. Taken together, these data demonstrate that new immunization strategies can markedly increase mucosal immunity in non-human primates and improve the protective efficacy against a mucosal respiratory virus challenge.

Previous studies of mucosal immunization with SARS-CoV-2 vaccines have largely focused on i.n. boosting strategies and have reported inconsistent increases in mucosal immunity^3–7^. Additional studies have shown that inhalational delivery was superior to i.n. delivery of an Ad5-TB vaccine in mice^30^ and that aerosol delivery was superior to i.m. delivery of an Ad5-TB vaccine for the induction of mucosal immune responses in humans^31^. An RSV vaccine has also been shown to be superior when delivered through the i.t. route compared with the i.n. route in baboons^32^. Moreover, a pharmacokinetic study in mice showed that i.n. administration of labelled nanoparticles resulted in only 28% reaching the lungs with substantial variability and most of the nanoparticles instead reached the stomach, whereas i.t. administration of nanoparticles resulted in 85–95% of the nanoparticles reaching the lungs, providing a potential explanation for the lack of consistency of i.n. boosting approaches^33^. Our data confirm and extend these previous observations by demonstrating that Ad26 i.t. boosting was substantially more potent than Ad26 i.n. boosting for nearly all of the immunological parameters tested. Moreover, we show that Ad26 i.t. boosting provided near-complete protection against the acquisition of infection after challenge with a high dose of SARS-CoV-2 BQ.1.1 in primates, which overcomes a key limitation of current SARS-CoV-2 vaccines that do not appear to induce robust mucosal immunity and do not protect against infection with current Omicron variants.

Vaccine delivery to the lungs in humans can be performed by inhalation and using nebulizer technologies^31^. The CanSino Ad5 vaccine has been approved in China through the inhalational route^34,35^, and the Bharat Biotech ChAd vaccine has been approved in India through the i.n. route, therefore demonstrating the clinical translatability of our findings. Our data comparing i.n. and i.t. immunization extend these observations and suggest that the delivery of the vaccine to the lungs is more effective than delivery of the vaccine to the nose. Moreover, our transcriptomics and cytokine data suggest that the mechanism of i.t. boosting involves robust and sustained activation of cytokine, NK, T and B cell pathways in the lungs.

The inability of the bivalent SARS-CoV-2 mRNA vaccines delivered through the i.m. route to provide robust protection against infection^1,2^ probably relates to their inability to induce robust mucosal immune responses at the portal of entry^10,11^. Our data demonstrate the proof-of-concept that mucosal boosting through the i.t. route results in robust mucosal humoral and cellular immune responses and near-complete protection against SARS-CoV-2 Omicron challenge in macaques. To the best of our knowledge, this degree of vaccine protection against SARS-CoV-2 Omicron challenge in macaques is qualitatively different from what has been reported previously with i.m.-delivered vaccines^19,29,36–38^. Adenovirus vectors may be particularly good at inducing mucosal immunity given their biophysical stability and natural mucosal tropism, although other vaccine platforms should also be explored as potential mucosal vaccines. Our data suggest that the development of next-generation vaccines that protect against infection with SARS-CoV-2^12,13^ and other respiratory viruses may be feasible by optimization of mucosal immunity.

## Methods

### Macaques and study design

In total, 40 outbred adult male and female rhesus macaques aged 4–8 years old previously received one or two i.m. primes with Ad26.COV2.S at weeks −114 and −108 and one boost i.m. with either Ad26.COV2.S or Ad26.COV2.S.351 (Beta) at week −69, as previously described^18^. All rhesus macaques were singly housed at Bioqual. Macaques that received two or three immunizations, and macaques that received Ad26.COV2.S or Ad26.COV2.S.351 at week −69 were equally divided into subsequent boosting groups. There were 3–4 female macaques and 2–3 male macaques in each group.

At week 0, macaques were boosted with 5 × 10^10^ viral particles of the bivalent Ad26.COV2.S + Ad26.COV2.S.529 (Omicron BA1) vaccine (Janssen/Johnson & Johnson) in 1 ml through the i.m. route, through the i.n. route using the mucosal atomization device (MAD; Teleflex) or through the i.t. route by direct tracheal inoculation by endoscopy or 30 μg of the lipid nanoparticle formulated bivalent mRNA vaccine (Pfizer-BioNTech; NIH SAVE Consortium) through the i.n. route using the MAD device (*n* = 6–7 macaques per group) (Fig. 1). Another group received no boost at week 0, and a sham (saline) control group was included. At study week 16, all of the macaques were challenged with 2 × 10^6^ PFU SARS-CoV-2 BQ.1.1 through the i.n. and i.t. routes in a total volume of 2 ml. The BQ.1.1 challenge stock (hCoV-19/USA/CA-Stanford-106_S04/2022, EPI_ISL_15196219) was produced in Vero-TMPRSS2 cells and had a titre of 8.25 × 10^6^ PFU per ml in VeroE6-TMPRSS2 cells and was fully sequence confirmed by M.S.S. After challenge, viral loads were assessed in the BAL and nasal swab samples using RT–qPCR analysis of *E* sgRNA. Macaques were euthanized on day 14 after challenge. Immunological and virological assays were performed in a blinded manner. All of the animal studies were conducted in compliance with all of the relevant local, state and federal regulations and were approved by the Bioqual Institutional Animal Care and Use Committee (IACUC).

### Pseudovirus NAb assay

NAb titres against SARS-CoV-2 variants used pseudoviruses expressing a luciferase reporter gene^23^. In brief, the packaging construct psPAX2 (AIDS Resource and Reagent Program), luciferase reporter plasmid pLenti-CMV Puro-Luc (Addgene) and spike-protein-expressing pcDNA3.1-SARS-CoV-2 SΔCT were co-transfected into HEK293T cells (ATCC, CRL_3216) with lipofectamine 2000 (Thermo Fisher Scientific). Pseudoviruses of SARS-CoV-2 variants were generated using the spike protein from WA1/2020 (Wuhan/WIV04/2019, GISAID accession ID: EPI_ISL_402124), BA.1 (GISAID ID: EPI_ISL_7358094.2), BA.5 (GISAID ID: EPI_ISL_12268495.2), BQ.1.1 (GISAID ID: EPI_ISL_14752457). The supernatants containing the pseudotype viruses were collected 48 h after transfection, and pseudotype viruses were purified by filtration with a 0.45 μm filter. To determine NAb titres in the serum, BAL fluid or nasal swab eluate, HEK293T-hACE2 cells were seeded in 96-well tissue culture plates at a density of 2 × 10^5^ cells per well overnight. Threefold serial dilutions of heat-inactivated serum samples, and twofold serial dilutions of heat-inactivated BAL and nasal swab samples were prepared and mixed with 60 μl of pseudovirus. The mixture was incubated at 37 °C for 1 h before adding to HEK293T-hACE2 cells. After 48 h, cells were lysed in Steady-Glo Luciferase Assay (Promega) according to the manufacturer’s instructions. SARS-CoV-2 neutralization titres were defined as the sample dilution at which a 50% reduction (NT_50_) in relative light units was observed relative to the average of the virus control wells.

### ELISA

SARS-CoV-2 spike receptor-binding domain (RBD)-specific binding antibodies in the serum, BAL fluid and nasal swab eluate were assessed by ELISA. Next, 96-well plates were coated with 1 μg ml^−1^ of SARS-CoV-2 WA1/2020, BA.1, BA.5 or BQ.1.1 RBD protein in 1× Dulbecco phosphate-buffered saline (DPBS) and incubated at 4 °C overnight. After incubation, the plates were washed once with wash buffer (0.05% Tween-20 in 1× DPBS) and blocked with 350 μl of casein block solution per well for 2 to 3 h at room temperature. After incubation, the block solution was discarded and plates were blotted dry. Serial dilutions of heat-inactivated serum, BAL fluid or nasal swab eluate were diluted in Casein block and added to wells, and the plates were incubated for 1 h at room temperature, before three more washes and a 1 h incubation with 1 μg ml^−1^ of anti-macaque IgG horseradish peroxidase (HRP) (NIH Nonhuman Primate Reagent Resource) or a 1:3,000 dilution of anti-monkey IgA HRP (Alpha Diagnostic) at room temperature in the dark. The plates were washed three times, and 100 μl of SeraCare KPL TMB SureBlue Start solution was added to each well; plate development was halted by adding 100 μl of SeraCare KPL TMB Stop solution per well. The absorbance at 450 nm, with a reference at 650 nm, was recorded using the VersaMax microplate reader (Molecular Devices). For each sample, the ELISA end-point titre was calculated using a four-parameter logistic curve fit to calculate the reciprocal serum dilution that yields a corrected absorbance value (450 nm–650 nm) of 0.2. Interpolated end-point titres were reported.

### ECLA

ECLA plates (MesoScale Discovery SARS-CoV-2 IgG, K15606U, panel 27; K15668U, panel 32 and IgA; K15608U, panel 27; and K15670, panel 32) were designed and produced with up to ten antigen spots in each well^25^. The plates were blocked with 50 μl of blocker A (1% BSA in distilled water) solution for at least 30 min at room temperature shaking at 700 rpm with a digital microplate shaker. During blocking the serum, diluted to 1:5,000 and BAL fluid, and nasal swab eluate was diluted 1:200 in Diluent 100. The plates were then washed three times with 150 μl of wash buffer (0.5% Tween-20 in 1× PBS), blotted dry and 50 μl of the diluted samples and calibration curve were added in duplicate to the plates and set to shake at 700 rpm at room temperature for at least 2 h. The plates were again washed three times and 50 μl of SULFO-Tagged anti-human IgG or anti-human IgA detection antibody diluted to 1× in Diluent 100 was added to each well and incubated with shaking at 700 rpm at room temperature for at least 1 h. The plates were then washed three times and 150 μl of MSD GOLD Read Buffer B was added to each well and the plates were read immediately after on the MESO QuickPlex SQ 120 machine. MSD titres for each sample are reported as relative light units, which were calculated as signal over background.

### Intracellular cytokine staining assays

CD4^+^ and CD8^+^ T cell responses were quantified using pooled peptide-stimulated intracellular cytokine staining assays^26^. Peptide pools contained 15 amino acid peptides overlapping by 11 amino acids spanning the SARS-CoV-2 WA1/2020, BA.1, BA.5 or BQ.1.1 spike proteins (21st Century Biochemicals). In total, 10^6^ BAL cells or PBMCs were resuspended in 100 µl of R10 medium supplemented with CD49d monoclonal antibodies (1 µg ml^−1^) and CD28 monoclonal antibodies (1 µg ml^−1^). Each sample was assessed with mock (100 µl of R10 plus 0.5% DMSO; background control), peptides (2 µg ml^−1^) and/or 10 pg ml^−1^ phorbol myristate acetate and 1 µg ml^−1^ ionomycin (Sigma-Aldrich) (100 µl; positive control) and incubated at 37 °C for 1 h. After incubation, 0.25 µl of GolgiStop and 0.25 µl of GolgiPlug in 50 µl of R10 was added to each well and incubated at 37 °C for 8 h and then held at 4 °C overnight. The next day, the cells were washed twice with DPBS, stained with aqua live/dead dye for 10 min and then stained with predetermined titres of monoclonal antibodies against CD279 (EH12.1, BB700), CD4 (L200, BV711), CD27 (M-T271, BUV563), CD8 (SK1, BUV805), CD45RA (5H9, APC H7) for 30 min. Cells were then washed twice with 2% FBS/DPBS buffer and incubated for 15 min with 200 µl of BD CytoFix/CytoPerm fixation/permeabilization solution. Cells were washed twice with 1× Perm Wash buffer (BD Perm/Wash Buffer 10× in the CytoFix/CytoPerm fixation/permeabilization kit diluted with MilliQ water and passed through a 0.22 µm filter) and stained intracellularly with monoclonal antibodies against Ki-67 (B56, BB515), IL-21 (3A3-N2.1, PE), CD69 (TP1.55.3, ECD), IL-10 (JES3-9D7, PE CY7), IL-13 (JES10-5A2, BV421), IL-4 (MP4-25D2, BV605), TNF (Mab11, BV650), IL-17 (N49-653, BV750), IFNγ (B27; BUV395), IL-2 (MQ1-17H12, BUV737), IL-6 (MQ2-13A5, APC) and CD3 (SP34.2, Alexa 700) for 30 min. Cells were washed twice with 1× Perm Wash buffer and fixed with 250 µl of freshly prepared 1.5% formaldehyde. Fixed cells were transferred to a 96-well round-bottom plate and analysed using the BD FACSymphony system. Data were analysed using FlowJo v.9.9.

### Subgenomic RT–qPCR assay

SARS-CoV-2 *E* gene sgRNA was assessed by RT–qPCR using primers and probes as previously described^23^. A standard was generated by first synthesizing a gene fragment of the subgenomic *E* gene. The gene fragment was subsequently cloned into the pcDNA3.1+ expression plasmid using restriction site cloning (Integrated DNA Technologies). The insert was in vitro transcribed to RNA using the AmpliCap-Max T7 High Yield Message Maker Kit (CellScript). log-transformed dilutions of the standard were prepared for RT–qPCR assays ranging from 1 × 10^10^ copies to 1 × 10^−1^ copies. Viral loads were quantified from the BAL and nasal swabs. RNA extraction was performed on the QIAcube HT system using the IndiSpin QIAcube HT Pathogen Kit according to manufacturer’s specifications (Qiagen). The standard dilutions and extracted RNA samples were reverse transcribed using the SuperScript VILO Master Mix (Invitrogen) according to the cycling conditions described by the manufacturer. A Taqman custom gene expression assay (Thermo Fisher Scientific) was designed using the sequences targeting the *E* gene sgRNA. The sequences for the custom assay were as follows, forward primer, sgLeadCoV2.Fwd, CGATCTCTTGTAGATCTGTTCTC; E_Sarbeco_R, ATATTGCAGCAGTACGCACACA; E_Sarbeco_P1 (probe), VIC-ACACTAGCCATCCTTACTGCGCTTCG-MGBNFQ. Reactions were performed in duplicate for samples and standards on the QuantStudio 6 and 7 Flex Real-Time PCR Systems (Applied Biosystems) with the following thermal cycling conditions: initial denaturation at 95 °C for 20 s; then 45 cycles of 95 °C for 1 s and 60 °C for 20 s. Standard curves were used to calculate sgRNA copies per ml or per swab. The quantitative assay sensitivity was determined as 50 copies per ml or per swab.

### Histopathology

Lungs from infected macaques were evaluated on day 14 after challenge at necropsy by histopathology. Blinded evaluation and histopathological scoring of four representative lung lobes from cranial, middle and caudal, left and right lungs from each monkey was performed by a board-certified veterinary pathologist (A.J.M.) with a scoring system that has been previously described^28^. At the time of fixation, the lungs were suffused with 10% formalin to expand the alveoli. All tissues were fixed in 10% formalin and blocks were sectioned at 5 μm. Slides were incubated for 30–60 min at 65 °C then deparaffinized in xylene and rehydrated through a series of graded ethanol to distilled water. Sections were stained with haematoxylin and eosin. For SARS-N immunohistochemistry, heat-induced epitope retrieval was performed using a pressure cooker on steam setting for 25 min in citrate buffer (Thermo Fisher Scientific, AP-9003–500), followed by treatment with 3% hydrogen peroxide. The slides were then rinsed in distilled water and protein blocked (Biocare, BP974M) for 15 min followed by rinses in 1× PBS. Primary mouse anti-SARS-CoV-nucleocapsid antibodies (Sinobiological; 40143-MM05) at 1:1,000 were applied for 60 min, followed by mouse Mach-2 HRP-Polymer (Biocare) for 30 min and the samples were then counterstained with haematoxylin followed by bluing using 0.25% ammonia water. Staining was performed using the Biocare intelliPATH autostainer. Nucleocapsid staining was negative (data not shown). Sirius red staining was performed to assess fibrosis.

### Bulk RNA-sequencing analysis in the BAL

BAL cells were lysed and extracted using the Quick-RNA MagBead kit (Zymo), which included DNase digestion. The RNA quality was assessed using the Bioanalyzer 2100 and TapeStation 4200 (Agilent) systems and then 10 ng of total RNA was used as an input for library construction using the SMARTer Stranded Total RNA-Seq Kit v2, Pico Input Mammalian (Takara Bio). Libraries were validated by capillary electrophoresis on a fragment analyser (Agilent), pooled at equimolar concentrations and sequenced with paired-end 100 bp reads on the Illumina NovaSeq 6000 system, yielding around 30 million reads per sample on average.

Alignment was performed using STAR v.2.7.9a and transcripts were annotated using a composite reference, including the Mmul10 assembly and annotation of the Indian rhesus macaque genome. Transcript abundance estimates were calculated internally to the STAR aligner using the algorithm of htseq-count. DESeq2 was used for normalization. Differential expression at the gene level between the different routes of vaccination and the no-boost group was performed using the raw count’s matrices by DESeq2 implemented in the DESeq2 R package. Adjusted *P* values were calculated by DEseq2 using Benjamini–Hochberg corrected of Wald test *P* values to assess significant genes upregulated or downregulated after immunization through three different routes compared with the no-boost group.

GSEA throughout the study was performed to assess enrichment in pathways. In brief, genes were preranked using the log_2_-transformed fold change in expression, and the enrichment of various genesets was tested after running 1,000 permutations of enrichment. MSigDB database C2 and the BTM modules were used to identify pathways that differentiated the Ad26 i.t., i.m. and i.n. groups compared with the no-boost group; a nominal adjusted *P*-value cut-off of 0.05 was used to assess the significance of these pathways. Leading-edge genes of significant pathways were selected for SLEA. SLEA was used to quantify the enrichment of each pathway within each sample. In brief, the expression of all of the genes in a specific pathway is averaged across each individual and compared to the average expression of 1,000 randomly generated gene sets of the same size. The resulting averaged expression was then scaled using *z* scores to reflect the overall perturbation of each pathway in each sample.

### Cytokine profiling in the BAL

ELISA (Mesoscale) serum and plasma cytokines and chemokines profiling was performed using the V-PLEX Plus NHP Cytokine 24-Plex Assay (K15058G-1, 2 plates, up to 52 samples) assay (Meso Scale MULTI-ARRAY Technology) commercially available by Meso Scale Discovery (MSD). A total of 300 μl of plasma or serum from each donor was combined with the biotinylated antibody plus the assigned linker and the SULFO-TAG-conjugated detection antibody; in parallel, a multianalyte calibrator standard was prepared by performing fourfold serial dilutions. Both samples and calibrators were mixed with the read buffer and loaded in a 10-spot V-PLEX plate, which was read by the MESOQuickPlex SQ 120 system. The samples were measured in duplicates. The plasma serum cytokines and chemokines values (pg ml^−1^) were extrapolated from the standard curve of each specific analyte. All values are given in pg ml^−1^ based on the calibration standard curve.

### Statistical analyses

Descriptive statistics and logistic regression were performed using GraphPad Prism v.9.0.0 (GraphPad Software). Immunological and virological data were generated in duplicate and pairwise comparisons were performed using two-sided Mann–Whitney *U*-tests. The primary objective of the study was to compare the protective efficacy of Ad26 i.t. versus Ad26 i.m. boosting against SARS-CoV-2 challenge, as measured by sgRNA levels in the BAL and nasal swabs after challenge. Correlations were assessed using two-sided Spearman’s rank correlation tests. A stepwise linear regression of sgRNA levels in the BAL and peripheral and mucosal immune responses was also performed using the Ad26 boost groups, and immune parameters were ranked according to their Spearman correlation. *P* *<* 0.05 was considered to be significant.

### Reporting summary

Further information on research design is available in the Nature Portfolio Reporting Summary linked to this article.

## Online content

Any methods, additional references, Nature Portfolio reporting summaries, source data, extended data, supplementary information, acknowledgements, peer review information; details of author contributions and competing interests; and statements of data and code availability are available at 10.1038/s41586-023-06951-3.

### Supplementary information

Reporting Summary

### Source data

Source Data Fig. 2

Source Data Fig. 3

Source Data Fig. 4

Source Data Fig. 5

Source Data Fig. 6

## Data Availability

All data are available in the Article. Transcriptomics data are available at the GEO (GSE245040). Source data are provided with this paper.
